# Comparative transcriptome analysis revealed the cooperative regulation of sucrose and IAA on adventitious root formation in lotus (*Nelumbo nucifera* Gaertn)

**DOI:** 10.1186/s12864-020-07046-3

**Published:** 2020-09-23

**Authors:** Cheng libao, Zhao minrong, Hu Zhubing, Liu huiying, Li Shuyan

**Affiliations:** 1 School of Horticulture and Plant Protection, Yangzhou University, Yangzhou, Jiangsu P. R. China; 2 College of Guangling, Yangzhou University, Yangzhou, Jiangsu P. R. China; 3 Key Laboratory of Plant Stress Biology, School of Life Sciences, Henan University, Kaifeng, Henan P. R. China

**Keywords:** Lotus, Adventitious roots, miRNA, Gene, Sucrose

## Abstract

**Background:**

In China, lotus is an important cultivated crop with multiple applications in ornaments, food, and environmental purification. Adventitious roots (ARs), a secondary root is necessary for the uptake of nutrition and water as the lotus principle root is underdeveloped. Therefore, AR formation in seedlings is very important for lotus breeding due to its effect on plant early growth. As lotus ARs formation was significantly affected by sucrose treatment, we analyzed the expression of genes and miRNAs upon treatment with differential concentrations of sucrose, and a crosstalk between sucrose and IAA was also identified.

**Results:**

Notably, 20 mg/L sucrose promoted the ARs development, whereas 60 mg/L sucrose inhibited the formation of ARs. To investigate the regulatory pathway during ARs formation, the expression of genes and miRNAs was evaluated by high-throughput tag-sequencing. We observed that the expression of 5438, 5184, and 5345 genes was enhanced in the GL20/CK0, GL60/CK0, and CK1/CK0 libraries, respectively. Further, the expression of 73, 78, and 71 miRNAs was upregulated in the ZT20/MCK0, ZT60/MCK0, and MCK1/MCK0 libraries, respectively. Kyoto Encyclopedia of Genes and Genomes (KEGG) pathway analysis revealed that most of the differentially expressed genes and miRNAs in the GL20/GL60 and ZT20/ZT60 libraries were involved in signal transduction. A large number of these genes (29) and miRNAs (53) were associated with plant hormone metabolism. We observed an association between five miRNAs (miR160, miR156a-5p, miR397-5p_1, miR396a and miR167d) and nine genes (auxin response factor, protein brassinosteroid insensitive 1, laccase, and peroxidase 27) in the ZT20/ ZT60 libraries during ARs formation. Quantitative polymerase chain reaction (qRT-PCR) was used to validate the high-throughput tag-sequencing data.

**Conclusions:**

We found that the expression of many critical genes involved in IAA synthesis and IAA transport was changed after treatment with various concentration of sucrose. Based on the change of these genes expression, IAA and sucrose content, we concluded that sucrose and IAA cooperatively regulated ARs formation. Sucrose affected ARs formation by improving IAA content at induction stage, and increased sucrose content might be also required for ARs development according to the changes tendency after application of exogenous IAA.

## Background

Lotus is an aquatic plant that is classified into three types, rhizome lotus (vegetable with rich nutrition), flower lotus (ornamental plant), and seed lotus (this plant can be treated as a kind of ornamental plant, and at the same time, the seed contains multiple nutrition). In China, lotus is commonly cultivated as an important off-season vegetable (long storage of product organ in soil) as the moderately humid climate provides the suitable growth conditions, especially in Yangtze River and Yellow River basin [1, 2]. In the last few decades, a variety of lotus products have been developed including lotus starch, lotus drink, and lotus tea. These products are exported to Korea, Japan and other Asian countries, which helps generate income to the local farmers [3]. Additionally, lotus is also used in traditional Chinese medicine to promote health.

As the principal root of lotus is underdeveloped, the major route for uptake of water and nutrition is through the adventitious roots (ARs) during the plant growth and development. The lotus ARs belong to the latent primordial form and often arises from the hypocotyl of the seedlings [4]. Meanwhile, a larger number of ARs (more than ten bouquets with 12-16 numbers in each bouquet) are developed at the internodes of the lotus rhizome when the storage organ is formed. Earlier studies have reported that ARs usually originate from the pericycle, in which the primordial root develops from the normal cell under certain conditions [5, 6]. The development of ARs includes the following stages: induction, initiation, and expression stages [7, 8]. Initially, the normal cells differentiate to form the meristematic cells, which form the root initials (sink establishment phase). Next, the primordium of the AR is established (the recovery phase) [9]. Finally, the AR primordium continues to develop until the AR break through the epidermis of the stem or leaf (the maintenance phase) [10]. Various internal (gene regulation or expression) and external factors (temperature, light, mechanical damage, hormone) are involved in the morphological and anatomical structure formation, physiological and biochemical or molecular regulation of the organ (including root) development [9, 11–13]. Therefore, the formation of AR is a heritable quantitative trait.

The plant biological processes including the germination of seed, and growth of seedlings, flower, and fruit are regulated by the endogenous factors, such as plant hormones, sugar, and peptides [14–16]. Sugars, such as sucrose can stimulate the root elongation [17]. Takahashi et al. 2003 [18] have reported that sucrose affects the AR formation at the induction stage and regulates adventitious root development similar to the plant hormones [19] However, there are no studies on the regulation of AR formation by sucrose through the modulation of hormone metabolism. The role of indole acetic acid (IAA) and ethylene in the formation of ARs has been previously reported [19–21]. The development of AR is affected by the ethylene metabolism or signal transduction, especially at the induction stage [22]. Treatment with 1-aminocyclopropane-1-carboxylic acid, an exogenous precursor of ethylene synthesis, markedly promotes the formation of ARs, which indicates that ethylene regulates AR development [23]. Similarly, IAA also has a role in the AR formation. An elevated level of IAA is observed to decrease the number of ARs. Conversely, low level of IAA accelerates the AR formation by affecting the cell division during the primordium formation [24]. Hence, AR formation is also dependent on the auxin signal transduction pathway. The synergistic interaction between ethylene and auxin is reported to regulate the process of AR formation [25]. Ethylene can also mediate the IAA-induced processes of AR by enhancing the IAA biosynthesis in the root [26]. Therefore, the formation of AR involves complex biological processes in plants.

Analyzing of gene regulation or expression is an efficient way to understand the formation of AR at the molecular level. Earlier studies suggested that some genes related to IAA metabolism (IAA transport or synthesis) play an important role during AR development [27]. There are two carrier proteins, the influx carrier and efflux carriers that are involved in IAA transport. The AUX influx carrier is known to be necessary for the IAA-regulated pathway of lateral roots formation [28]. PIN, an efflux carrier is also involved in the lateral root development [29]. PIN exhibits tissue-specific expression with its expression observed in the primordial tissue [30]. Additionally, the genes with LOB domain motif are required for the AR development. The expression of these genes is known to be induced by IAA or ethylene. *ARL1* is reported to be involved in the AR development during the induction stage by promoting cell dedifferentiation, which is regulated by IAA [31]. Further, microRNAs (18-24 nucleotide in length) are also known to affect the plant growth by regulating the functional gene expression [32, 33]. Many developmental processes including stress-response or adaptation, and organ formation are regulated by the microRNAs [34, 35]. Some auxin responsive factors such as ARF17, ARF6, and ARF8 are regulated by miR160 and miR167 during AR development in *Arabidopsis* [36]. Hou et al. (2019) report that the overexpression of miR171 and miR390 in tomato plant can increase the root number compared to the wild type plants [37]. Therefore, microRNA is believed to be an important regulator of plant growth and development.

Lotus is commonly propagated asexually in production. In lotus breeding, asexual propagation is used to generate desirable traits by hybridization and to select the variations. The lotus ARs are important for plant growth as the principal root of lotus is undeveloped. Therefore, analyzing the regulatory pathway at the molecular level is necessary for exploring the mechanisms underlying AR formation.

## Results

### The effect of sucrose on lotus ARs formation

**Fig. 1:**
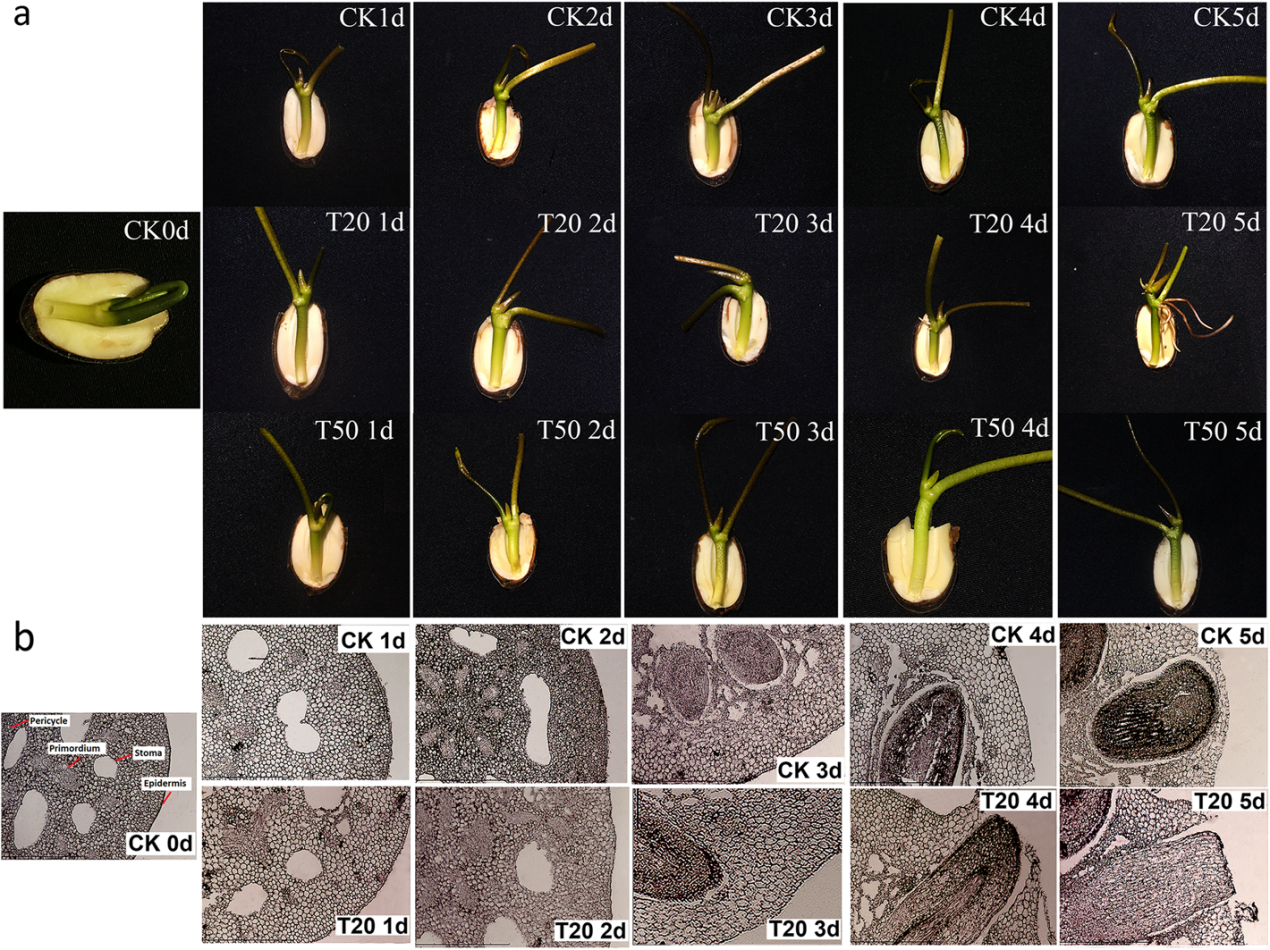
The changes of morphology and microstructure of ARs after treatment of sucrose. **a** The morphology change of ARs in lotus seedlings after 20 mg/L and 60 mg/L sucrose treatment within 5 days (T20 or T60 represented seedlings treated with 20 mg/L or 60 mg/L sucrose for 1-5 d, CK represented the seedling growing in the water for 1-5 d). **b** The microstructure changes of the ARs in lotus seedlings after 20 mg/L sucrose treatment within 5s days by paraffin section (T20 represented seedlings treated with 20 mg/L sucrose for 1-5 d, CK represented the seedlings growing in the water for 1-5 d)

To evaluate the role of sucrose on AR formation, we treated the lotus seedlings with 20 mg/L or 60 mg/L sucrose for 2 days. We observed that the two-day treatment with these concentrations of sucrose was enough to affect the formation of AR. Further, the effect of 20 mg/L sucrose treatment was different from that of 60 mg/L sucrose treatment on AR development. There was a significant improvement in AR formation upon treatment with 20 mg/L sucrose compared to the untreated group. Meanwhile, the AR did not emerge from the epidermis of seedling hypocotyl upon treatment with 60 mg/L sucrose before 5 days (Fig. 1a). As there was no formation of AR upon treatment with 60 mg/L sucrose, we only analyzed the microstructure of hypocotyl that was treated with 20 mg/L sucrose. We observed that some AR primordiums were distributed around the stomata in the germinated seed, which suggested that AR development was latent primordial form. Further, we also observed that the ARs on the hypocotyl was induced (induction stage) after treatment with 20 mg/L sucrose for 1-2 days. The untreated plant needed approximately 3-4 days to complete this process. Additionally, the primordium near the pericycle (inner primordium) was induced earlier than that near the epidermis. The AR of the treated seedlings began to break through the epidermis after 3-4 days, while the same in control plant took approximately 5 days. This suggested that 20 mg/L sucrose promoted the development of epidermis (Fig. 1b).

### Identification of genes and small RNAs involved in AR formation

**Fig. 2:**
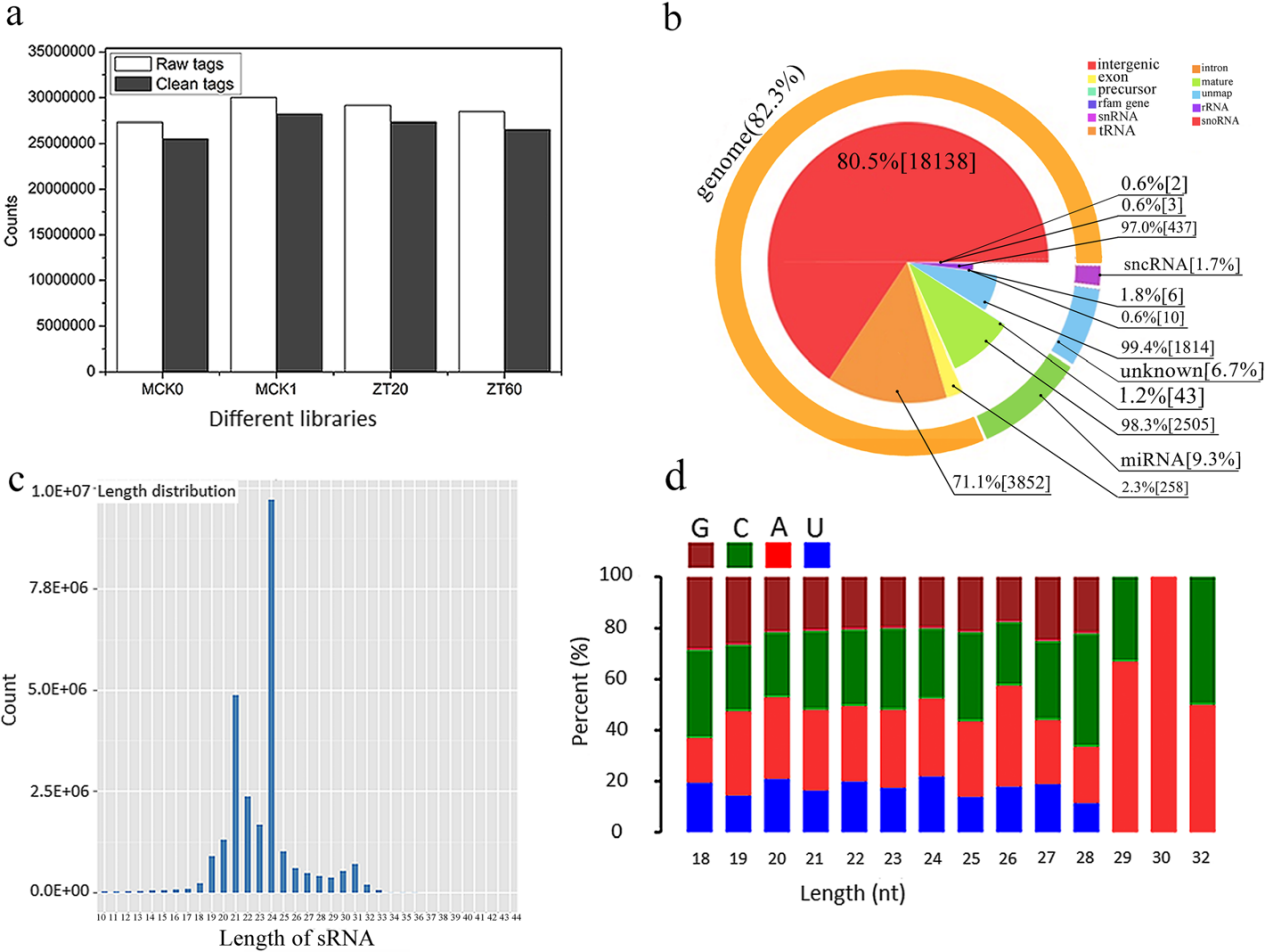
The detailed information of miRNAs sequenced by RNA-seq technology. **a** the number of raw tags or clean tags in four libraries of ARs (MCK0 represented the tags number in the germinated stage, MCK1, ZT20 or ZT20 represented the tags number in the seedlings after water, 20 mg/L or 60 mg/L sucrose treatment for 1 d respectively. **b** The proportion of all kinds of small RNA after comparison against known sRNA database. **c** The number of small RNAs in different distribution length of small RNAs. **d** Statistics of the first base of predicted miRNAs with 18-32 nucleotides

The metabolic change in plants is regulated by the small RNA-mediated degradation of mature RNA. We constructed four libraries for evaluating the gene expression (CK0, CK1, GL20, and GL60 libraries), and four libraries for evaluating the miRNA expression (MCK0, MCK1, ZT20, and ZT60 libraries). The dataset generated by RNA-seq was able to achieve 100% lotus genome coverage, which was evident from the flat curve of the identified gene numbers with increasing sequencing depth (Additional file 1: Fig. S1). Additionally, we observed a high correlation value among the replicates in each sample (Additional file 1: Fig. S2). We obtained more than 2.1 × 10^7^ reads (approximately 99% of clean reads) for gene expression and 2.7 × 10^7^ reads (approximately 93% of clean reads) for miRNA expression after the low quality reads were removed (Fig. 2a, Additional file 1: Table S2).

Among the four miRNA libraries, we analyzed the sequence data of only one library as there was no significant difference in the dataset between the four miRNA libraries. We observed that a large number (80.5%) of clean reads obtained from RNA-seq were classified as the intergenic sequence, and only 9.3% of reads were classified as the miRNA sequence (Fig. 2b). The length of the miRNA sequences was in the range of 18-32 nucleotides with a predominant sequence length of 24 nucleotide (Fig. 2c). The further analysis of 18-32 nucleotides revealed that the least and highest number of nucleotides were uracil and adenine, respectively (Fig. 2d).

### Differentially expressed genes and miRNAs in the libraries

**Fig. 3:**
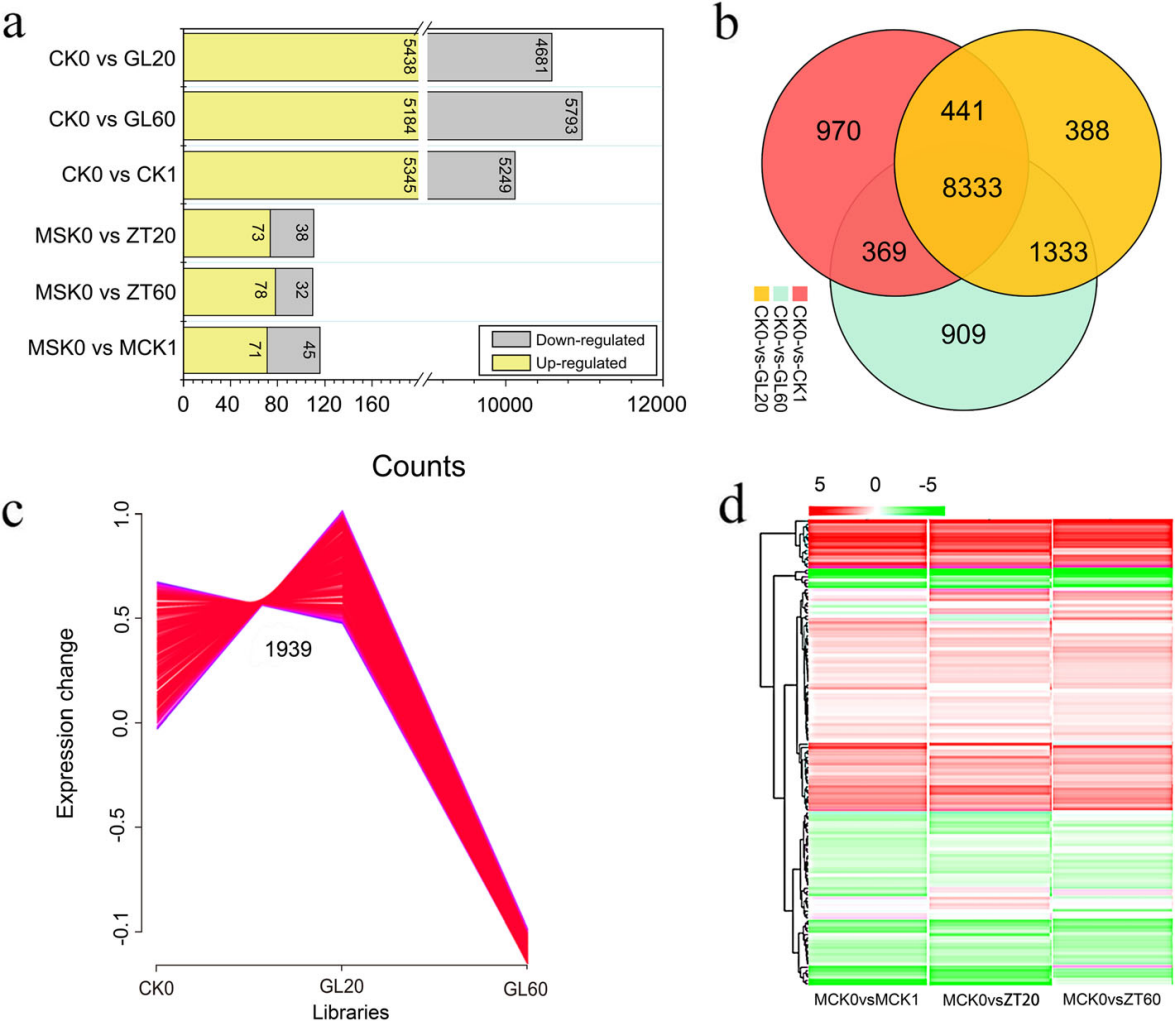
The expression change of mRNAs or miRNAs after treatment with 20 mg/L and 60 mg/L sucrose. **a** The changed number of mRNAs or miRNAs after treatment with 20 mg/L and 60 mg/L sucrose. **b** The expression of genes involved in CK1/CK0, GL60/CK0 and GL20/CK0 libraries. **c** The most desirable expression profile in CK0, GL20 and GL60 libraries. **d** the fold change of miRNAs in MCK1/MCK0, ZT60/MCK0 and ZT20/MCK0 libraries

Based on the identification of differentially expressed genes and miRNAs, we constructed 4 libraries each for genes and miRNAs that are involved in the development of AR. We observed that a total of 5438, 5184, and 5345 genes were upregulated, and 4681, 5793, and 5249 genes were downregulated in the GL20/CK0, GL60/CK0, and CK1/CK0 libraries, respectively. Additionally, an enhanced expression of 73, 78, and 71 miRNAs and a decreased expression of 38, 32, and 45 miRNAs were observed in the ZT20/MCK0, ZT60/MCK0, and MCK1/MCK0 libraries, respectively (Fig. 3a, Additional file 1: Tables S3, S4). We also observed that 388, 909, and 970 genes were specifically expressed between the CK0 and GL20 stages, the CK0 and GL60 stages, and the CK0 and CK1 stages, respectively (Fig. 3b). Among the differentially expressed genes, we observed a total of twelve types of gene expression profiling, and an enhanced expression of 1936 genes was observed in the GL20/CK0 libraries, which was worthy of further study (Fig. 3c, Additional file 1: Fig. S3). Meanwhile, most genes in the GL20/CK0, GL60/CK0 and CK1/CK0 libraries exhibited less ten-fold change in expression (Additional file 1: Fig. S4). Similarly, the miRNAs in the MZT20/MCK0, MZT60/CK0, and MCK1/CK0 libraries exhibited less than ten-fold change in expression (Fig. 3d, Additional file 1: Fig. S5).

### KEGG pathway analysis of the differentially expressed genes and miRNAs

**Fig. 4:**
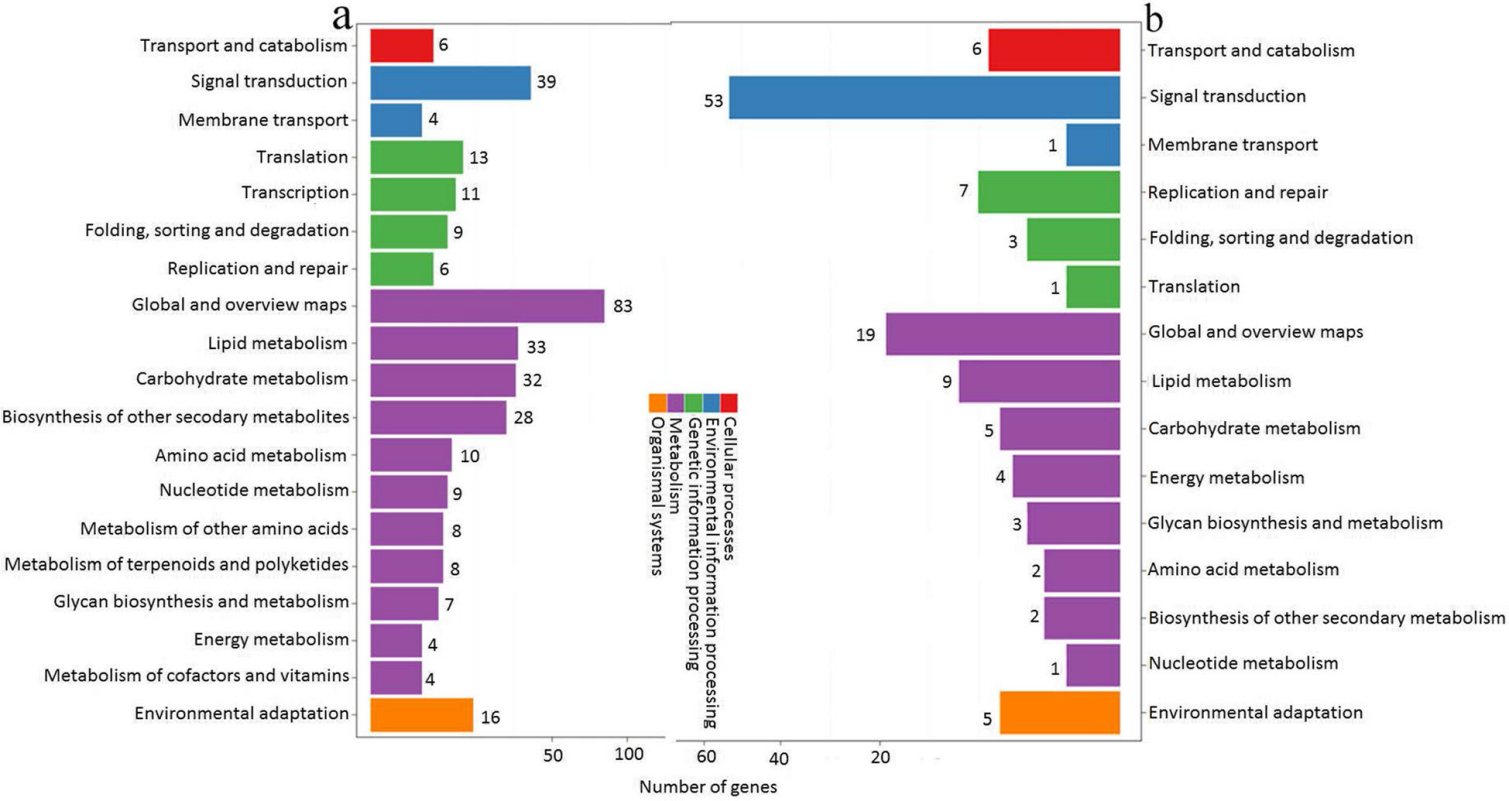
KEGG classification of differentially expressed mRNAs or miRNAs in GL20/GL60 and ZT20/ZT60 libraries. The X axis showed the number of differentially expressed mRNAs or miRNAs, and the Y axis showed the second KEGG pathway terms. The first pathway terms were indicated using different colors, and second pathway terms were subgroups of the first pathway terms, and were grouped together on the X axis on the right side. **a** The number of differentially expressed mRNAs in GL20/GL60 libraries. **b** The number of differentially expressed miRNAs in ZT20/ZT60 libraries

**Fig. 5:**
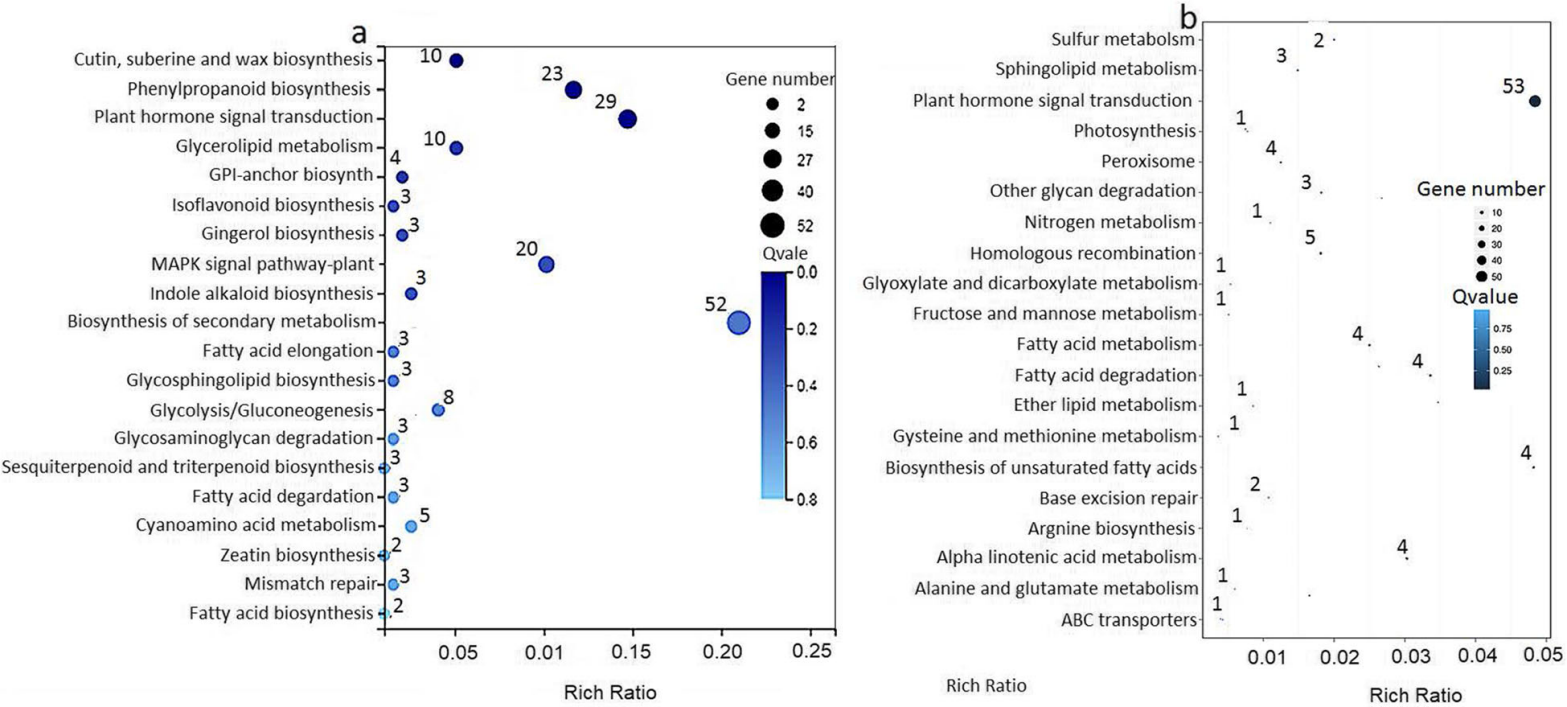
The display of the top 20 enriched pathway terms in GL20/GL60 and ZT20/ZT60 libraries. The rich factor was the ratio of differentially expressed mRNAs or miRNAs numbers annotated in this pathway term to all gene numbers annotated in this pathway term, and the greater the rich factor, the greater the degree of enrichment. **a** The display of the top 20 enriched pathway terms in GL20/GL60 libraries. **b** The display of the top 20 enriched pathway terms in ZT20/ZT60 libraries

We annotated the differentially expressed genes and miRNAs in the G20/G60 and ZT20/ZT60 libraries during AR development using the KEGG tool. All the differentially expressed genes and miRNAs were classified into five groups: cell processes, environmental information processing, genetic information processing, metabolism, and organismal systems. In case of gene expression libraries, we observed that the most pathway changes were associated with global and overview maps (83 genes with altered mRNA level), followed by signal transduction (39 differentially expressed genes) in the G20/G60 libraries (Fig. 4a). Additionally, the largest number (53 miRNAs) of miRNAs that were differentially expressed belonged to the signal transduction pathway in the ZT20/ZT60 libraries (Fig. 4b). Among the 39 genes and 53 miRNAs involved in signal transduction, 29 genes and 53 miRNAs were involved in the plant hormone signal transduction, especially IAA metabolism or signaling (Fig. 5, Tables 1 and 2). This suggested that IAA metabolism or signaling has a role in the lotus AR formation.

**Table 1:** The differentially expressed genes involved in plant hormone metabolism in GL20/GL60 libraries

Gene	log_**2**_(GL20/GL60)	Q-value	***P***-value	Kegg Orthology
LOC104602697	5.16	0.0457	0.0000372	Aprataxin
LOC104607466	5.16	0.00152	0.0000372	SAUR family protein
LOC104607586	3.80	0.0000187	1.52e-20	Auxin influx carrier (AUX1 LAX family)
LOC104599117	2.57	6.13e-68	7.53e-65	Xyloglucan:xyloglucosyl transferase
LOC104588219	2.44	0.0000134	4.22e-32	Aaprataxin
LOC104593190	2.02	2.15e-10	2.93e-11	Glucurono kinase interconversion
LOC104599121	1.97	0	1.96e-30	Xyloglucan:xyloglucosyl transferase TCH4
LOC104599116	1.96	2.86e-24	0.000125	Xyloglucan:xyloglucosyl transferase TCH4
LOC104605544	1.87	1.21e-64	9.01e-12	Auxin influx carrier (AUX1 LAX family)
LOC104608184	1.84	0.0987	0.0000546	Protein brassinosteroid insensitive 1
LOC104590115	1.64	6.52e-14	3.34e-28	Auxin response factor
LOC104595568	1.63	2.88e-12	0.0000129	Glucuronokinase
LOC104599115	1.57	0	8.66e-32	Xyloglucan:xyloglucosyl transferase TCH4
LOC104600761	1.53	2.39e-34	3.81e-10	Abscisic acid receptor PYR/PYL family
LOC104604490	1.53	1.27e-10	0.0000368	Histidine-containing phosphotransfer peotein
LOC104599114	1.48	0	1.04e-20	Xyloglucan:xyloglucosyl transferase
LOC104604448	1.47	7.43e-25	3.33e-12	Aprataxin
LOC104609806	1.11	1.20e-34	0.00000137	Potassium channel
LOC104599122	1.10	0.000149	0.00000201	Xyloglucan:xyloglucosyl transferase TCH4
LOC104589695	1.09	6.73e-24	0.0000639	SAUR family protein
LOC104589702	1.05	1.25e-8	5.65e-9	SAUR family protein
LOC104607475	1.03	4.18e-7	0.00000504	Auxin response factor
LOC104606136	1.02	3.94e-83	7.47e-11	Auxin responsive GH3 gene family
LOC104600857	1.01	1.5e-27	0.000847	Ethylene-responsive transcription factor 1
LOC104585739	−1.02	8.46e-9	2.11e-12	DELLA protein
LOC104606659	−1.22	0	4.27e-240	Pathogenesis-related protein 1
LOC104606713	−1.26	0	0.0000677	Transcription factor MYC2 + ko04016

**Table 2:** The differentially expressed miRNAs involved in plant hormone metabolism in ZT20/ZT60 libraries

miRNAs	Description
miR156a-5p	Tquamosa promoter-binding-like protein 3 isoform
miR156a-5p	Squamosa promoter-binding-like protein 6 isoform
miR160a-5p	Auxin response factor 17-like
miR156a-5p	Squamosa promoter-binding-like protein 3 isofor
miR171b-3p	Scarecrow-like protein 6
miR156a-5p	Squamosa promoter-binding-like protein 7
miR156a-5p	Squamosa promoter-binding-like protein 18
miR156a-5p	Squamosa promoter-binding-like protein 14
miR156a-5p	Squamosa promoter-binding-like protein 6
miR156a-5p	Squamosa promoter-binding-like protein 13A
miR160a-5p	AUX/IAA protein
miR156a-5p	Squamosa promoter-binding-like protein 18
miR156a-5p	Squamosa promoter-binding-like protein 12
miR393a-5p	F-box domain
miR156a-5p	Squamosa promoter-binding-like protein 12
miR156a-5p	Squamosa promoter-binding-like protein 13A
miR156a-5p	Squamosa promoter-binding-like protein 13A
miR156a-5p	Squamosa promoter-binding-like protein 13A
miR156a-5p	Squamosa promoter-binding-like protein 6
miR393a-5p	Protein transport inhibitor response 1
miR156a-5p	Squamosa promoter-binding-like protein 6
miR156a-5p	Squamosa promoter-binding protein 1
miR156a-5p	Squamosa promoter-binding-like protein 18
miR156a-5p	Squamosa promoter-binding-like protein 6
miR156a-5p	Squamosa promoter-binding-like protein 6
miR156a-5p	Squamosa promoter-binding-like protein 3
miR156a-5p	Squamosa promoter-binding protein 1
miR171b-3p	Scarecrow-like protein 22
miR156a-5p	Squamosa promoter-binding-like protein 18
miR156a-5p	Squamosa promoter-binding-like protein 6
miR156a-5p	Squamosa promoter-binding-like protein 13A
miR156a-5p	Squamosa promoter-binding-like protein 6
miR171b-3p	Scarecrow-like protein 15
miR156a-5p	Squamosa promoter-binding-like protein 3
miR156a-5p	Squamosa promoter-binding-like protein 13A
miR156a-5p	Squamosa promoter-binding-like protein 3
miR160a-5p	Auxin response factor 18
miR5368	Shaggy-related protein kinase eta
miR160a-5p	Auxin response factor 17
miR156a-5p	Teosinte glume architecture 1
miR156a-5p	Squamosa promoter-binding-like protein 3
miR156a-5p	Squamosa promoter-binding-like protein 6
miR156a-5p	Squamosa promoter-binding-like protein 17
miR393a-5p	F-box domain
miR393a-5p	F-box domain
miR156a-5p	Squamosa promoter-binding-like protein 18
miR156a-5p	Teosinte glume architecture 1
miR156a-5p	Squamosa promoter-binding-like protein 13A
miR160a-5p	Auxin response factor 17
miR156a-5p	Squamosa promoter-binding-like protein 13A
miR156a-5p	Teosinte glume architecture 1
miR156a-5p	Squamosa promoter-binding-like protein 18
miR156a-5p	Squamosa promoter-binding-like protein 18

### Association analysis of genes and miRNAs related to AR development

**Fig. 6:**
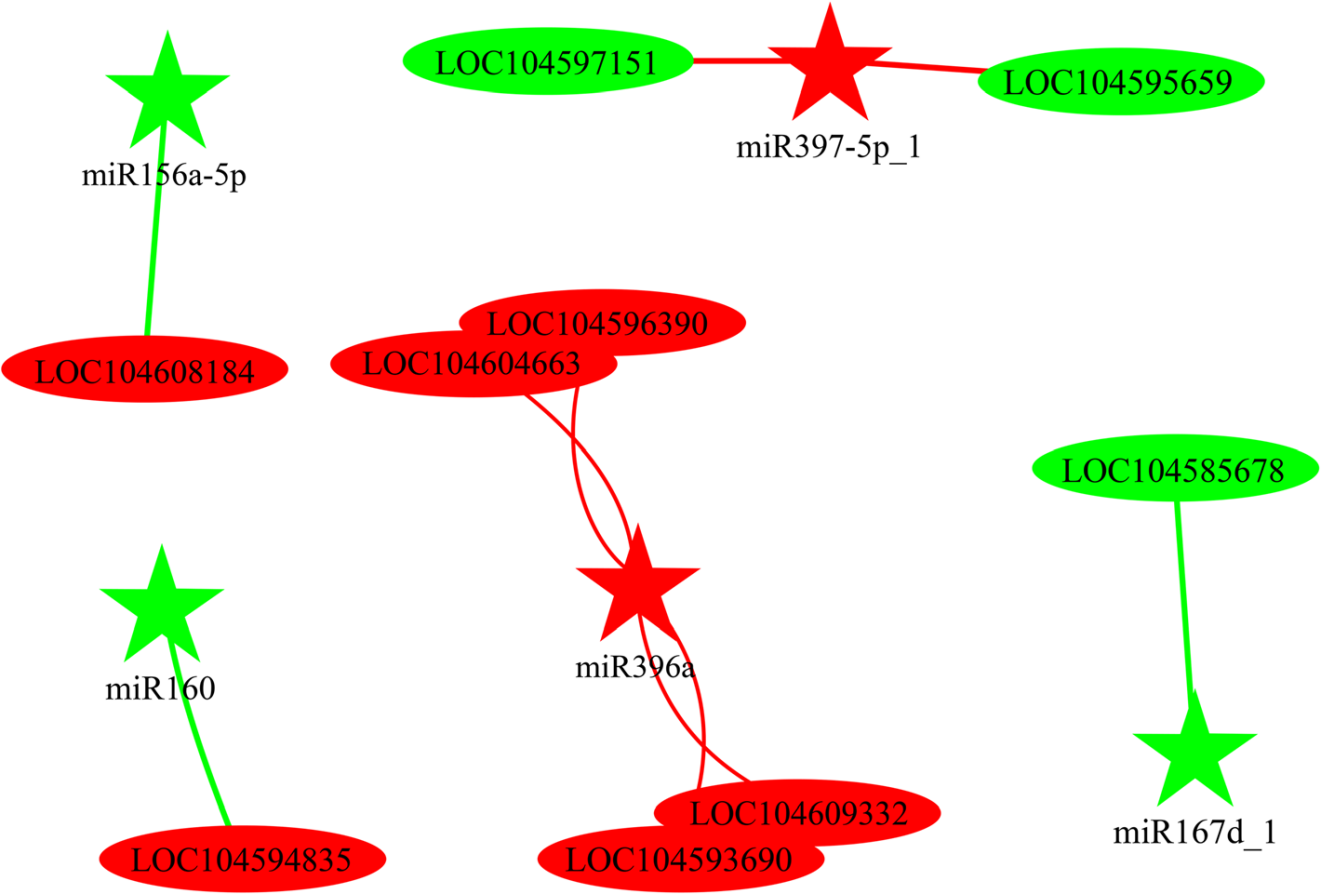
Associated analysis of miRNAs and mRNAs in ZT20/ZT60 libraries. The red and the green ellipse representing up-regulated or down-regulated genes. The red and green five-pointed star representing up-regulated genes or miRNAs or down-regulated miRNAs. LCO 104608184 was protein brassinosteroid insensitive 1; LCO104594385 was auxin response factor 17; LCO104595695 and LOC104597151 were Laccase; LCO104585678 was peroxidase 27; LCO104596390, LCO104604663, LCO1045 93,690 and LCO104609332 were lysine-specific demethylase 3

We used the association analysis to evaluate the correlation between differentially expressed genes and miRNAs during AR development. We obtained 121 pairs of genes and miRNAs (95 pairs exhibited negative regulation and 26 pairs exhibited positive regulation) in the CK1/CK0 libraries. Similarly, we obtained 122 pairs of genes and miRNAs in the GL20/CK0 libraries (99 pairs exhibited negative regulation and 23 pairs exhibited positive regulation). In the GL60/CK0 libraries, 98 pairs of genes and miRNAs exhibited negative regulation and 23 pairs exhibited positive regulation with high correlation (Additional file 1: Table 5). Based on these data derived from RNA-seq, we further analyzed the correlation of genes and miRNAs between the GL20/GL60 and ZT20/ZT60 libraries. The analysis revealed that five miRNAs could regulate nine genes (mRNAs) during the AR formation. Among these 5 miRNAs, miRNA396a positively regulated four genes, and miRNA397-5P negatively regulated two genes in the GL20/GL60 libraries (Fig. 6, Table 3).

**Table 3:** Associated analysis of genes and miRNAs (including negative regulation and positive regulation) in ZT20/ZT60 libraries

miRNA ID	Target ID	miRNA foldchange	Target foldchange	Description
ZT20/ZT60 libraries				
Negative regulation				
miR160	XR_734793.2	−2.28	3.24	Auxin response factor
miR156a-5p	XM_010274087.1	−1.12	1.61	Protein brassinosteroid insensitive 1
miR397-5p_1	XM_010258572.2	1.02	−1.12	Laccase
miR397-5p_1	XM_019197470.1	1.02	−1.01	Laccase
Positive regulation				
miR396a	XM_010253658.2	1.13	1.01	Lysine-specific demethylase 3
miR396a	XM_010257521.2	1.13	1.02	Lysine-specific demethylase 3
miR396a	XM_010269107.1	1.13	1.08	Lysine-specific demethylase 3
miR396a	XM_010275619.2	1.13	6.39	Lysine-specific demethylase 3
miR167d	XM_010242633.2	−3.38	−1.71	Peroxidase 27

### IAA and sucrose content identification and qRT-PCR analysis

The germinated seeds were treated with sucrose (20 mg/L and 60 mg/L) and IAA (10 μmol/L and 150 umol/L) for 2 days and were allowed to proceed with development in water. The IAA and sucrose content was quantified at day 0, 2, 4, 6 post-treatment for IAA, and 0, 2, 4, 6, and 8 for sucrose. We observed that the IAA content in the seedlings that were treated with 20 mg/L sucrose was higher than that in the seedlings treated with 60 mg/L sucrose or the untreated seedling at day 0. At day 2 post-treatment, the highest IAA content was also observed in the 20 mg/L sucrose treated seedlings, followed by untreated seedlings. The seedlings treated with 60 mg/L sucrose had the least IAA content within 6 d of post treatment. The IAA content in all the three groups decreased at day 4 post-treatment (Additional file 1: Fig, S6). This indicated that sucrose affect the IAA synthesis at the induction stage, which directly promoted the development of AR. In addition, it was showed that the seedlings treated with 10 umol/L IAA and untreated seedlings showed higher sucrose content than that in the seedlings treated with 150 umol/L IAA at day 0 ~ 4 post-treatment, although no significant difference was found between 10 umol/L IAA treated and untreated seedlings. The seedlings treated with 10 umol/L IAA had the highest content at day 4 ~ 8 post-treatment. The seedlings treated with 150 umol/L IAA had the lowest sucrose content within 8 d after treatment.

**Fig. 7:**
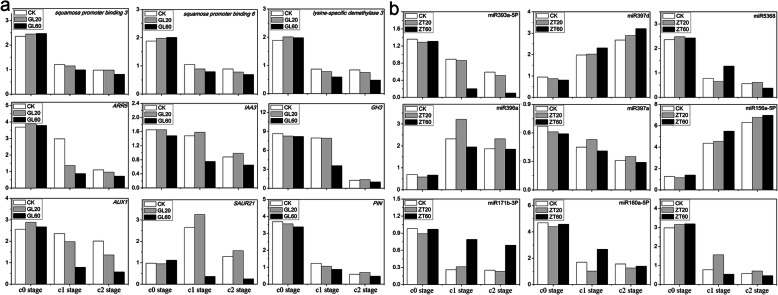
The expression of genes and miRNAs in C0, C1 and C2 stages after treatment of 60/L (GL60 or ZT60) and 20 mg/L (GL20 or ZT20) sucrose. **a** The expression of nine genes by qRT-PCR technology in in C0, C1 and C2 stages; **b** The expression of nine miRNAs by qRT-PCR technology in C0, C1 and C2 stages

We selected 9 differentially expressed genes (SAUR21, GH3, auxin responding factor 5, auxin influx carrier, lysine-specific demethylase 3, squamosa promoter binding like protein 6, squamosa promoter-binding-like protein 3, PIN, and scarecrow like protein 15) and 9 differentially expressed miRNAs (miR393a-5P, miR160a, miR156a-5p, miR160a-5p, miR171b-3p, miR5368, miR396a, miR157d, and miR397a_3) to analyze their expression during different developmental stages of AR (C0 stage, C1 stage, and C2 stage) by qRT-PCR. The expression pattern of these genes evaluated by qRT-PCR was similar to that evaluated by RNA-seq. Therefore, the result obtained from sequencing was reliable (Fig. 7).

### Genes and miRNAs involved in IAA metabolism and signal transduction pathway

**Fig. 8:**
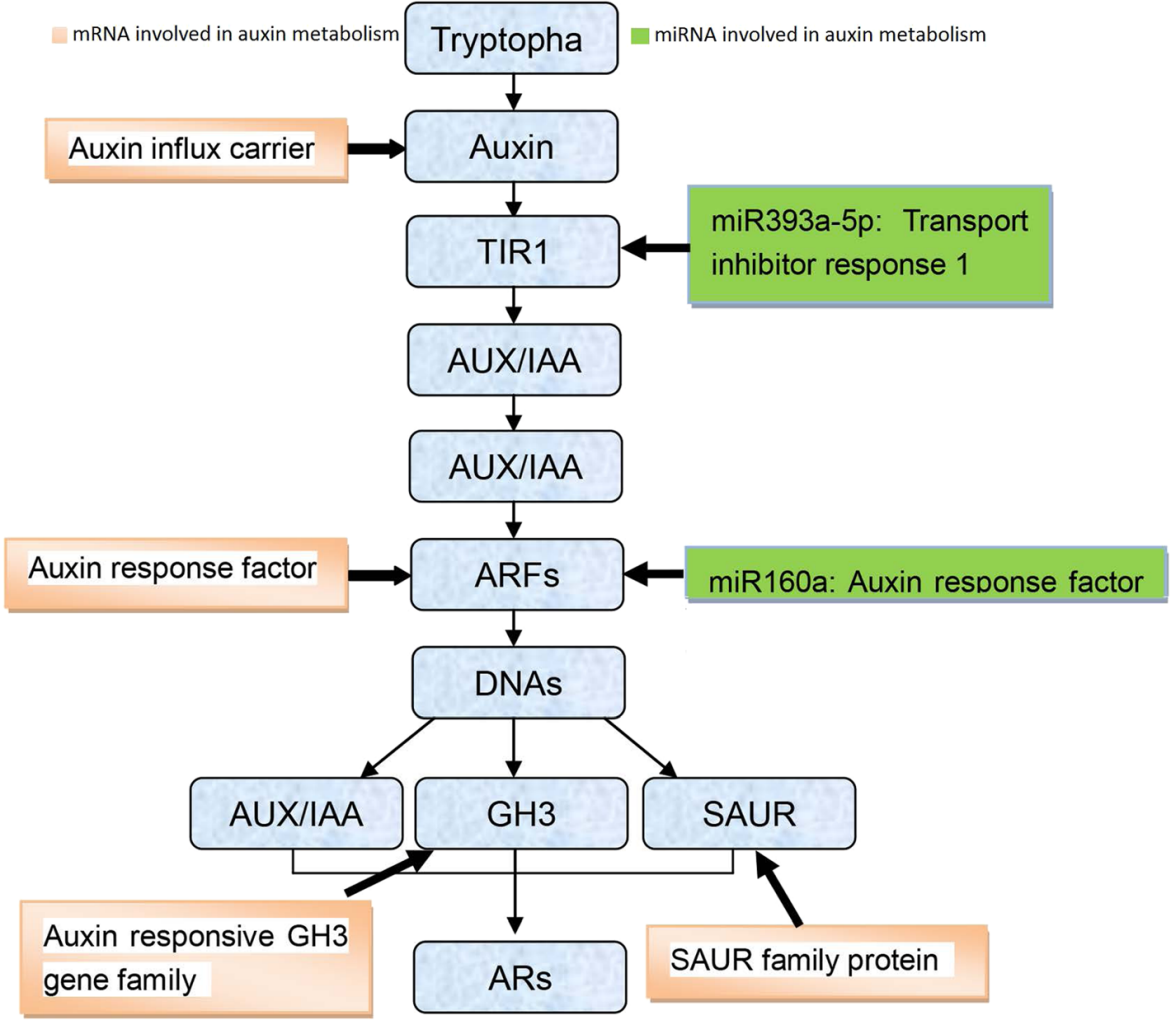
Genes or miRNAs involved in auxin metabolism. Yellow rectangle repressed genes involved in auxin metabolism, and green rectangle repressed miRNAs involved in auxin metabolism. Blue rectangle repressed the processes of auxin metabolism

A large number of differentially expression genes and miRNAs are associated with IAA metabolism or signaling. Hence, we evaluated the change in expression of all the genes and miRNAs upon treatment with different concentrations of sucrose during AR formation. We observed that four different types of genes including auxin influx carrier, auxin responding factor, auxin responsive GH3 gene family and SAUR, and two miRNAs (miR393a-5P and miR160a) were involved in IAA pathway. Meanwhile, we also observed the expression of nine genes of peroxidase (five up-regulated genes or four down-regulated genes), which may regulate the IAA content, was affected for miRNA level (Additional file 1: Table S6). Above results suggested that most differentially expressed genes and miRNAs affected various biological processes in the IAA pathway. Only the ARF pathway was common for the cooperative participation of genes and miRNAs. Auxin responding factor, which is regulated by miR160a were important associated network to affect the AR development in lotus (Fig. 8).

## Discussion

### Analysis of genes and miRNAs sequenced by RNA-seq technique

As the principal roots are not developed in lotus, ARs are required to regulate the plant growth and development [4, 38]. Lotus is an important aquatic plant that has several applications such as food, products, ornamental plant, water purification and medicine. The process of water and nutrition uptake is completed only by the ARs in lotus. The formation of ARs can be classified into three stages: induction of root primordium, development of root primordium, and the emergence of ARs from the hypocotyl [38]. These three stages are regulated by genetic and environmental factors [4, 9].

### The role of IAA on the formation of ARs

Plant hormone metabolism or signaling is known to play a crucial role in most of the metabolic processes. We identified various differentially expressed genes and miRNAs that were associated with IAA metabolism or signaling during AR formation post-sucrose treatment, ased on these data and the results reported earlier [4]. In this study, four libraries during lotus ARs formation in responding to sucrose were constructed to monitor metabolism change based on lotus seedlings develpmental feature (Fig. 1a, b) sequenced by solexa technology, which has been widely applied in exploring mechanism of plant growth and development [39, 40]. Wehypothesized that the plant hormone, IAA plays a crucial role during AR formation. Therefore, we evaluated the expression of genes related to IAA synthesis, transport, or degradation. We observed that the indole-3-acetic acid-amido synthetase, GH3 gene in the sucrose-treated group exhibited 0.36, − 0.17, and − 1.87 fold change in expression in the CK1/CK0, GL20/CK0 and GL60/CK0 libraries, respectively, compared to the untreated group. However, in the sucrose-treated group there was a 1.87 fold increase in the expression of GH3 in the GL20/GL60 libraries compared to the untreated group (Additional file 1: Table S3). This suggested that the change in the expression of IAA synthesis-related genes between two concentrations of sucrose might have a differential effect on AR formation. Therefore, our result indicated that exogenous sucrose affected ARs development by improving IAA content at induction stage (by improving gene expression related with IAA metabolism) Theactivity of peroxidase is also affected by the treatment with exogenous indolebutyric acid during AR development [41], which suggests a high correlation between IAA content and peroxidase. It is hypothesized that the change in peroxidase expression may affect the IAA content by oxidative decarboxylation reaction [42]. Among the nine peroxidase genes evaluated in this study, five genes were upregulated and four genes were downregulated in the GL20/GL60 libraries (Additional file 1 Table S6). However, further studies are needed to understand the correlation between peroxidase and endogenous IAA content in this process. IAA transport is involved in the root formation in plant kingdom. Auxin is believe as an influx carrier to transport IAA from the synthetic organ to the other developmental tissue [43]. A marked decrease in the number of lateral roots is observed when the expression of *AUX1* is silenced [44]. The role of auxin has been reported to affect the AR development at the early stages of lateral root primordium [28]. In this study, an enhanced expression of *auxin* was observed in the GL20/GL60 libraries. This may be the reason for the differential response observed in the 20 mg/L and 60/L sucrose treatment groups. However, upon sucrose treatment we observed a down-regulation of *auxin* expression by 0.39 fold, − 1.89 fold, and − 5.87 fold in the CK1/CK0,GL20/CK0, and GL60/CK0 libraries, respectively (Addition file 1 Table S3). PIN, an efflux carrier, transports IAA through the concentration and electrochemical gradients in plants [45–47]. Reinhardt et al. (2003) reported an abnormal root formation in the *atPIN* mutant of *Arabidopsis* [48]. The constitutive expression of *osPIN* in rice distinctly promotes development of root in the transgenic plant, which suggests that *osPIN* is required for the development of ARs in rice [30]. These studies have demonstrated that *PIN* is an important factor for the development of ARs. In this study, we observed an enhanced expression of *PIN7* in the GL20/GL60 libraries. Additionally, *PIN7* expression in the sucrose-treated group changed by − 3.05 fold, − 3.8 fold, and − 5.0 fold in the CK1/CK0, GL20/CK0, and GL60/CK0 libraries, respectively during AR formation (Additional file 1: Table S3). The expression profile was similar to that of *AUXI*. However, the decreased expression of *PIN7* in the GL20/CK0 libraries was less than that observed in the GL60/CK0 libraries. Leyser (2001) reported that auxin affects AR development by inducing the root primordial formation which is completely dependent on auxin signal transduction [49]. *PLD2* plays an important role in the auxin signal transduction. The auxin signal transduction is inhibited in *PLD2* knockout plants [8, 50]. However, we observed a similar pattern of enhanced phospholipase D expression in the CK1/CK0,GL20/CK0, and GL60/CK0 libraries (Additional file 1: Table S3), this suggested that the response to different concentrations of sucrose during AR development was not due to the *PLD2* expression during the induction stage.

### miRNA regulation involved in auxin signal transduction during root development

MicroRNA (miRNA) plays an important role on AR formation by regulating auxin synthesis or by inducing the expression of auxin response factors. Previously, some miRNAs such as miR1670, miR160, miR172, miR167, miR164, and miR393 have been reported to be involved in the root development [51]. The regulating miRNAs such as miR160-*MbARF16,* miR164-*MbNAC1* and miR393-*MbTIR1* have been reported to affect the root formation [52]. The gene encoding indole glucosinolate biosynthetic enzyme is involved in the IAA synthesis and is regulated by miR10515 [53]. Similarly, osAFB, a member of auxin signaling transmitter, which is involved in root development is regulated by miR393 [54]. Therefore, the regulatory network of AR development is very complex, which involves various genes and regulators. In this study, five models of gene regulation were observed (Fig. 8). The negative regulating models (miRNA156a-5P-LCO104608184, miRNA397-5p-LOC104597151, miRNA-5p-LOC104595695 and miRNA160a-LOC104596385) were found to show high correlation suggested that these genes along with miRNAs played an important role for the formation of ARs. Specifically, the miRNA160a-LOC104596385 warrants further study.

## Conclusions

We demonstrated that while high concentration of sucrose (60 mg/L) markedly inhibited AR formation, low concentration of sucrose (20 mg/L) promoted the AR development in lotus. We studied the expression of genes and miRNAs in four libraries: CK0, CK1, GL20, and GL60 libraries for gene expression; MCK0, MCK1, ZT20, and ZT60 libraries for miRNA expression. We obtained more than 2.1 × 10^7^ reads in both gene expression libraries and miRNA expression libraries. A total of 5438, 5184, and 5345 genes were upregulated, and 4681, 5793, and 5249 genes were downregulated in the GL20/CK0, GL60/CK0, and CK1/CK0 libraries, respectively. Additionally, an enhanced expression of 73, 78, and 71 miRNAs and a decreased expression of 38, 32, and 45 miRNAs were observed in the GL20/CK0, GL60/CK0, and CK1/CK0 libraries, respectively. In the GL20/GL60 and ZT20/ZT60 libraries, a total of 29 genes and 53 miRNAs were associated with the plant hormone signal transduction, especially IAA metabolism or signaling. Further, five miRNAs and nine genes had a high correlation in the GL20/GL60 libraries with the AR formation. Some genes and miRNAs involved in IAA metabolism or signal transduction were also analyzed.

## Methods

### Plant growth and sample preparation

In this study, the “Taikong 36” lotus was used for microstructure, RNA sequencing (RNA-seq), and quantitative real-time polymerase chain reaction (qRT-PCR) analyses. The rhizome (“seed”) was grown in the open field of Yangzhou University, Southeast China with conventional management in early April. During the growth season (from early April to mid-October), the depth of water was maintained at 20-40 cm. The average temperature during the growth season was maintained at 25-29 °C during day and 20 °C during night. The lotus seeds were collected in autumn, and placed in the storage house at normal temperature.

### Preparation of paraffin sections

The lotus seeds were treated with 20 mg/L sucrose. The growth conditions for the seedlings were similar as described above. The hypocotyls from the treated or control group were collected at six time points (day 0, 1, 2, 3, 4, and 5 post-treatment). The hypocotyls were firstly cut into 3 mm × 3 mm × 2 mm (length × width × height) sections. The sections were then transferred into a small bottle with free fatty acid fixing fluid (at least twenty times the volume of fixing samples). A syringe was used to form vacuum within the bottle with fixing samples for 5 s, and the cap was opened to exchange gas for 5 min. This was repeated thrice, and the bottle was placed into a normal temperature overnight. The sample was treated with 50, 70, 85, 95% and absolute ethanol for 30 min sequentially. The samples were first treated with a mixed solution (pure xylene: absolute ethanol; 1:1), followed by treatment with pure xylene for 30 min. The paraffin debris was added into the bottle, and incubated at temperature overnight. The paraffin blocks were prepared by placing the tissues into the thawed paraffin wax for 18-24 h. The wax tape with a thickness of 10 μm was cut using a slicer, and placed on a glass slide. The glass slide with wax tape was transferred into pure xylene, mixed solution (pure xylene: absolute ethanol; 1:1) and absolute ethanol sequentially for 5-10 min. The slide was air-dried and the tissue was observed under an optical microscope.

### Library construction and sequencing of genes and miRNAs

For RNA-seq analysis, the seed coat of the lotus seed was broken to allow water absorption, and placed into a container with a water depth of 5 cm at 28-30 °C to allow germination. The germinated seeds (approximately 2 days of germination) were treated with 20 mg/L and 60 mg/L sucrose for 2 days, and transferred into water for allowing growth. The samples were collected at day 0 (for C0 or MCK0 library construction) and day 1 (for the construction of CK1, GL20 and GL60 libraries for gene expression analysis; for the construction of MCK1, ZT20 and ZT60 libraries for miRNA expression analysis) post-treatment with 20 mg/L and 60 mg/L sucrose. The RNA was extracted from the hypocotyls at room temperature and was treated with purified DNaseI to degrade the DNA. We used approximately 1 μg RNA from each sample for the library construction. For the construction of CK0, CK1, GL20 and GL60 libraries, the samples were parallelly evaluated using Illumina gene expression sample preparation kits. The detailed protocol is previously described by Cheng et al. (2018) [4]. For miRNA expression analysis (MCK0, MCK1, ZT20 and ZT60 libraries), the RNA sample was resolved to obtain different sizes by polyacrylamide gel electrophoresis (PAGE), and 18-30 nucleotide (14-30 ssRNA Ladder Marker, TAKARA) sequence was selected. We prepared the 3′ and 5′ adaptors using the Tru Seq Small RNA Sample Pre Kit, Illumina, following the following protocols of Cheng et al. (2019) [55]. The special construct was prepared by Beijing Institute of Genomics (BIG).

### Screening for differentially expressed gene and small miRNAs (DESs)

The screening for differentially expressed genes (DEGs) was performed as described previously [56, 57]. The resulting reads were used for the downstream analysis with NOISeq method as described by Cheng et al. (2018) [4]. The gene expression in samples of each group was expressed as log_2_ (fold-change) M and the absolute value of difference (D) of all pair conditions was calculated to build the noise distribution model. For gene A, NOISeq computed as its average expression in control group (control_average) and average expression in treatment group (treat_average). The fold change (MA = log2 ((treat_avg)/(control_avg))) and the absolute value of difference D (DA = |control_avg - treat_avg|) were calculated. If MA and DA diverged from the noise distribution model markedly, then gene A was defined as DEG and DES. There was a probability value to assess the divergence of MA and DA from the noise distribution model. Only those genes with a fold change ≥2 and divergence probability ≥0.8 were considered as DEGs and DESs.

### Annotation of differentially expressed genes and small miRNAs

The genes and miRNAs obtained in this experiment were annotated using the Gene Ontology (GO) tool with three ontologies such as molecular function, cellular component, and biological process. All the DEGs and DESs were enriched and classified into various biological functions after comparison with the lotus genome obtained from the National Center for Biotechnology Information (NCBI) database. All the DESs were compared to the GO database (http://www.geneontology.org/), and the number of genes and miRNAs were calculated for the three ontologies mentioned above. Further, these differentially expressed genes and miRNAs were then input into a list of significantly enriched GO terms by hypergeometric test. For pathway (biological functions) analysis, KEGG tool was applied for the organize enrichment analysis of DEGs and DESs. Therefore, all the DEGs and DESs were grouped into different metabolic pathways.

### qRT-PCR analysis

We analyzed the expression of some genes and miRNAs to monitor the change in metabolism after sucrose treatment under the cultivation conditions described above. The mRNA expression of nine genes and miRNAs at three time points (C0 stage: germinated seeds, C1 stage: day 1 post-treatment, C2: day 3 post-treatment) was quantified after treatment with 20 mg/L or 60 mg/L sucrose. The gene expression was analyzed using quantitative polymerase chain reaction (qPCR) as described previously [12, 58]. The total RNA from the plant was extracted using the RNA extraction mini kit (QIAGEN, Germany). The RNA was treated with DNaseI to remove the DNA contamination. cDNA synthesis was performed with 2-3 μg of RNA using the First Strand cDNA Synthesis Kit (Fermentas, USA) [59]. The mRNA expression levels was measured in triplicates using SYBR Green Master Mix (Tiangen, China) on the Mx 3000P machine (STRATAGENE, http:// www.stratagene.com) [60]. The PCR primer for the genes and miRNAs was designed by primer 5.0 software based on the sequences obtained from the NCBI database or RNA-seq data, and the detailed information of primers was listed in Additional file 1 Table S1. β-Actin was used as the internal standard. The primers used in the study were: upstream, 5′-AACCTCCTCCTCATCGTACT-3′, and downstream, 5′-GACAGCATCAGCCATGTTCA-3′. The reaction volume for the PCR was 25 μL, which consisted of 12.5 μL SYBR Premix Ex Taq II (TliRNaseH Plus) (2X), 10 μM each of the forward and reverse primers, 2 μL cDNA solution, and 8.5 μL distilled water. The PCR condition was 94 °C for 30 s, followed by 40 cycles of 95 °C for 5 s and 50-60 °C for 60 s. The data analysis was carried out by 2^-△△Ct^ method. △Ct value was obtained according to the Ct_(target)_ and Ct_(actin)_ value in treated plants (△Ct_(target)_) and control (△Ct_(normal_), and △△Ct value was counted based on the data of △Ct_(target)_ and △Ct_(normal)_. Therefore, 2^-△△Ct^, which represented the relative expression level was determined.

### IAA and sucrose determination

The IAA quantification was performed following the method of Pence et al. (2013) [61]. The seed coat of the lotus seeds was broken and the seeds were germinated in the water at normal temperature. The germinated seeds were treated with 20 mg/L and 60 mg/L sucrose for 2 days, and transferred into water to allow growth. We collected 50 treated and control seedlings at day 0, 2, 4, and 6 for estimation of IAA. The hypocotyl was powdered, and 1-2 g of the powder was placed into 10 mL of 0.01 M phosphate buffer (8 mM K_2_HPO_4_, 2.7 mM KCl, 135 mM NaCl and 1.5 mM KH_2_PO_4_, pH 6.5) with sufficient mixing. The suspension was left undisturbed at room temperature for 10 min. The mixture was centrifuged at 12000 rpm for 10-15 min. The supernatant was collected and subjected to ELISA to quantify the IAA content. The germinated seeds were treated with 10 μmol/L and 150 μmol/L IAA (10 μmol/L IAA significantly promoted ARs formation, and while 150 μmol/L distinctly inhibited ARs development) for 2 days, and transferred into water for continue growth. We collected 50 treated and control seedlings at day 0, 2, 4, 6 and 8 for estimation of sucrose. The detailed steps was refered as Wight et al. (1983) [62].

## Supplementary information

**Additional file 1: Fig. S1.** Analysis of sequencing data saturation in CK0, CK1, GL20 and GL60 libraries.a. C0 library. b.CK1 library. c. GL20 library. d. GL60 library. **Fig. S2.** The correlation of gene expression between samples, especially for three repeated samples in CK0, CK1, GL20 and GL60 libraries respectively. **Fig. S3.** The patterns of gene expression in CK0, GL20 and GL60 libraries. Genes with similar expression pattern in those stages were classified into one cluster. **Fig. S4.** Fold change of gene expression in different libraries. Red points represented up-regulated DEGs, blue points represented down-regulated DEGs, and gray points represented non-DEGs. a. Gene expression in CK1/CK0 libraries. b.Gene expression in GL20/CK0 libraries. c. Gene expression in GL60/CK0 libraries. **Fig. S5.** Fold change of gene expression in different libraries. Red points represented up-regulated miRNAs, blue points represented down-regulated miRNAs, and gray points represented no change of miRNAs. a. miRNAs expression in CK1/CK0 libraries. b.miRNAs expression in GL20/CK0 libraries. c. miRNAs expression in GL60/CK0 libraries. **Fig. S6.** Identification of IAA and sucrose content during ARs Formation. a. determination of IAA content at 0 d, 2 d, 4 d and 6 d after treatment of 20 g/L and 60 g/L sucrose in the lotus seedlings. b. Identification of sucrose content at 0 d, 2 d, 4 d, 6 d and 8 d after treatment of 10 μmol/L and 150 μmol/L IAA in the lotus seedlings. **Table S1.** The primers of genes and miRNAs used for expression analysis by qRT-PCR technology. **Table S2.** The detailed information of tags in every library of gene or miRNA sequenced by RNA-seq technology. **Table S3.** Differentially expressed genes in CK1/CK0, GL20/CK0 and GL60/CK0 libraries. **Table S4.** Differentially expressed miRNAs in MCK1/MCK0, ZT20/MCK0, ZT60/MCK0, ZT20/MCK1 and ZT60/MCK1 libraries. **Table S5.** Association analysis of different expressed genes and miRNAs in MCK1/MCK0, ZT20/MCK0 and ZT60/MCK0 libraries. **Table S6.** The change of gene expression related with peroxidases in ZT20/ZT60 libraries.

## Data Availability

The materials of all the experiment was supported by aquatic vegetable Lab of Yangzhou University. The collection of seed complied with local and national guidelines and permissions of seed were obtained. The detail data has been deposited in NCBI database (Biosample number, CK0_1 ~ GL60_3: SAMN11581378 ~ SAMN11581389, MCK0_1 ~ ZT60_3: SAMN11571191 ~ SAMN11571202; Bioproject number, CK0_1 ~ GL60_3: PRJNA541277, MCK0_1 ~ ZT60_3: PRJNA541047).
