# Supplementary material for: Comparative transcriptome analysis revealed the cooperative regulation of sucrose and IAA on adventitious root formation in lotus (Nelumbo nucifera Gaertn)
Source: BMC Genomics. 2020 Sep 23;21:653. doi: 10.1186/s12864-020-07046-3 (PMC7510093; doi:10.1186/s12864-020-07046-3)
Supplement: Supplementary file 1 — Additional file 1: Fig. S1. Analysis of sequencing data saturation in CK0, CK1, GL20 and GL60 libraries.a. C0 library. b.CK1 library. c. GL20 library. d. GL60 library. Fig. S2. The correlation of gene expression between samples, especially for three repeated samples in CK0, CK1, GL20 and GL60 libraries respectively. Fig. S3. The patterns of gene expression in CK0, GL20 and GL60 libraries. Genes with similar expression pattern in those stages were classified into one cluster. Fig. S4. Fold change of gene expression in different libraries. Red points represented up-regulated DEGs, blue points represented down-regulated DEGs, and gray points represented non-DEGs. a. Gene expression in CK1/CK0 libraries. b.Gene expression in GL20/CK0 libraries. c. Gene expression in GL60/CK0 libraries. Fig. S5. Fold change of gene expression in different libraries. Red points represented up-regulated miRNAs, blue points represented down-regulated miRNAs, and gray points represented no change of miRNAs. a. miRNAs expression in CK1/CK0 libraries. b.miRNAs expression in GL20/CK0 libraries. c. miRNAs expression in GL60/CK0 libraries. Fig. S6. Identification of IAA and sucrose content during ARs Formation. a. determination of IAA content at 0 d, 2 d, 4 d and 6 d after treatment of 20 g/L and 60 g/L sucrose in the lotus seedlings. b. Identification of sucrose content at 0 d, 2 d, 4 d, 6 d and 8 d after treatment of 10 μmol/L and 150 μmol/L IAA in the lotus seedlings. Table S1. The primers of genes and miRNAs used for expression analysis by qRT-PCR technology. Table S2. The detailed information of tags in every library of gene or miRNA sequenced by RNA-seq technology. Table S3. Differentially expressed genes in CK1/CK0, GL20/CK0 and GL60/CK0 libraries. Table S4. Differentially expressed miRNAs in MCK1/MCK0, ZT20/MCK0, ZT60/MCK0, ZT20/MCK1 and ZT60/MCK1 libraries. Table S5. Association analysis of different expressed genes and miRNAs in MCK1/MCK0, ZT20/MCK0 and ZT60/MCK0 libraries. Table S6. Th [file 12864_2020_7046_MOESM1_ESM.zip › Additional file 1 Table. S1.docx]

| Name | Up-stream (5´-----3´) | Down-stream (5´-----3´) |
| --- | --- | --- |
| Gene´s primers | | |
| SAUR21 | CACCGATTTATCCACCGTTCTTG | GTGCTTGATCCCGGCGATTGTAT |
| Indole-3-acetic acid-amido synthetase GH3 | GTACATCCCGACGCTCAAGCACTA | ATGTTGGGCATCAGGGTGTAGGAC |
| Squamosa promoter binding like protein 6 | AGTCTTGGAGCTATGGTTCTGAAGG | CGACTCTGAGCTAACATGGTGCTT |
| Squamosa promoter binding like protein 3 | GGAATCCAAAGACTCCCTCACAG | ACCACCTCCCGCAGATGAATAGAC |
| IAA33 | TGACACCACGACACCTTTCATACC | ACGTCCCTCTAGCACAACCGTAA |
| Lysine-specific histone demethylase 1 | CTATGTTGACCACGGAGGGATTA | AGACAAGCATAAGCAGGCGAACA |
| Auxin efflux carrier component 6 | CGGACCTACAGTAATGTCAGCAGC | TAGTAGAGGAGGGTGACAGGCAAG |
| Auxin efflux carrier family protein | GGCGAATACTCTGGTTCCCTCAT | AAACAATGGAAGCAGCCGTCTCA |
| Auxin response factor 5-like | CATTGCACCAGATCACCTTACCC | GCTGTTTAGGTTGTTTGCGTCCA |
| miRNA´s primers |  |  |
| miR397a_3 | CGGTTTCGACAGCGCTGCACT |  |
| miR157d | CGCGCGTGACAGAAGATAGAGAGCAC |  |
| miR396a | CGGCGCTTCCACAGCTTTCTTGAACTG |  |
| miR5368 | CGGCGGGACAGTCTCAGGTAGACA |  |
| miR171b-3p | AGCGTTGAGCCGTGCCAATATCACG |  |
| miR160a-5p | TGCCTGGCTCCCTGTATGCCA |  |
| miR156a-5p | CGGCGTGACAGAAGAGAGTGAGCAC |  |
| miR160a | cgTGCCTGGCTCCCTGAATGCT |  |
| miRNA393a | TACTGCGACTGGAGTAG |  |
